# Bilateral effects of unilateral cerebellar lesions as detected by voxel based morphometry and diffusion imaging

**DOI:** 10.1371/journal.pone.0180439

**Published:** 2017-07-10

**Authors:** Giusy Olivito, Michael Dayan, Valentina Battistoni, Silvia Clausi, Mara Cercignani, Marco Molinari, Maria Leggio, Marco Bozzali

**Affiliations:** 1 Ataxia Laboratory, Santa Lucia Foundation, Rome, Rome, Italy; 2 Neuroimaging Laboratory, Santa Lucia Foundation, Rome, Italy; 3 Pattern Analysis and Computer Vision, Istituto Italiano di Tecnologia, Genova, Italy; 4 Department of Psychology, "Sapienza" University of Rome, Rome, Italy; 5 Clinical Imaging Sciences Centre, Brighton and Sussex Medical School, Falmer, United Kingdom; 6 Neurological and Spinal Cord Injury Rehabilitation, Department A, Santa Lucia Foundation, Rome, Italy; 7 Department of Neuroscience, Brighton and Sussex Medical School, University of Sussex, Falmer, United Kingdom; Mathematical Institute, HUNGARY

## Abstract

Over the last decades, the importance of cerebellar processing for cortical functions has been acknowledged and consensus was reached on the strict functional and structural cortico-cerebellar interrelations. From an anatomical point of view strictly contralateral interconnections link the cerebellum to the cerebral cortex mainly through the middle and superior cerebellar peduncle. Diffusion MRI (dMRI) based tractography has already been applied to address cortico-cerebellar-cortical loops in healthy subjects and to detect diffusivity alteration patterns in patients with neurodegenerative pathologies of the cerebellum. In the present study we used dMRI-based tractography to determine the degree and pattern of pathological changes of cerebellar white matter microstructure in patients with focal cerebellar lesions. Diffusion imaging and high-resolution volumes were obtained in patients with left cerebellar lesions and in normal controls. Middle cerebellar peduncles and superior cerebellar peduncles were reconstructed by multi fiber diffusion tractography. From each tract, measures of microscopic damage were assessed, and despite the presence of unilateral lesions, bilateral diffusivity differences in white matter tracts were found comparing patients with normal controls. Consistently, bilateral alterations were also evidenced in specific brain regions linked to the cerebellum and involved in higher-level functions. This could be in line with the evidence that in the presence of unilateral cerebellar lesions, different cognitive functions can be affected and they are not strictly linked to the side of the cerebellar lesion.

## 1. Introduction

Over the past two decades, the role of the cerebellum in cognition has been widely demonstrated [1–4]. Anatomically, cerebello-cortical-cerebellar connections are known to be strictly controlateral and to be spatially and functionally organized in distinct parallel loops [5–6]. The afferent system consists of cortico-pontine fibers projecting from cerebral cortex areas to the pontine nuclei and of ponto-cerebellar fibers, crossing the midline to enter the cerebellum by means of the contralateral Middle Cerebellar Peduncle (MCP) [7]. Conversely, the Superior Cerebellar Peduncle (SCP) is well known to be the efferent fibers system from the cerebellum [8–9] decussating at the level of the midbrain and projecting to motor and associative cortices via the thalamus [5–6, 10]. This complex neural system allows the cerebellum to receive, optimize and send back the information that it receives from cerebral cortex regions to accomplish motor and cognitive functions successfully. Functional studies with healthy subjects also support the anatomical evidence of functionally related parallel cortico-cerebellar loops [11]. Based on recent evidence from a Voxel Based Morphometry (VBM) study, the clinical alterations consequent to a focal cerebellar lesion are associated with specific structural modifications in cerebello-related areas of the cerebral cortex [12]. The interruption of cerebello-thalamo-cortical pathways has been reported as the mechanism responsible for crossed cerebello-cerebral diaschisis (CCCD) [13– 15]. In this context, the functional impairment in the cerebral regions contralateral to the cerebellar lesion has been explained as a functional depression of cerebello-ponto-thalamo-cerebral pathways [13]. Thus, it is possible to hypothesize that a disruption of this pathway is responsible for the functional depression of those cerebral regions from where a motor or cognitive command originates, to reach the cerebellum which in turn redistributes new cerebellar-processed information back to the same cerebral regions. Since MCP and SCP are the feedback and feedforward limbs of the cerebello-cortical system it is reasonable to think that cerebellar white matter (WM) alterations, secondary to the presence of cerebellar damage, may affect the cerebello-cortical interaction and result in hypoactivity of supratentorial brain regions accounting for the various clinical dysfunctions typically observed [16–17]. It follows that investigating cerebellar white matter microstructure is required to understand the cerebello-cortical alterations subtending the complex cerebellar cognitive affective syndrome [3]. Diffusion Tensor Imaging (DTI) has proven to be a valuable tool for investigating brain white matter since it can probe tissue microstructure by assessing the displacement of water molecules within specific WM tracts [18]. Although diffusion-derived indices provide a very indirect measure of microstructural properties, they have been associated with specific white matter abnormalities. Among them, Radial Diffusivity (RD) has been shown to be positively correlated with fiber disruption [19–20]. Thanks to its ability to reconstruct 3-dimensional fiber bundles (a process known as tractography), DTI also appears relevant in providing a model of brain connectivity through which brain disconnection can be studied. Recently, the ability of DTI tractography to map and quantify the whole trajectory of different cortico-cerebellar pathways has been demonstrated in normal adult brain [8, 21–22] as well as in patients with ataxia and cerebellar tremor [23]. A probabilistic atlas of cerebellar WM has been recently proposed contributing to a better understanding of cerebellar WM architecture [17]. One well-known limitation of DTI tractography is the inherent assumption of a single fiber direction per voxel, which limits its applicability to white matter areas of crossing-fibers. In order to compensate for this limitation a number of higher order models of diffusion have been introduced (see Alexander, 2005 for a review[24]), and applied recently to reconstruct cerebellar peduncles [25].

The microstructural organization of cerebellar WM tracts has never been investigated in patients affected by focal cerebellar damage. Isolated cerebellar lesions have been reported to produce an impairment of cognitive performances. Verbal, executive and visuospatial abilities can be selectively affected based on lesion lateralization and distribution [4]. In light of these assumptions, patients with isolated cerebellar lesions represent an interesting model to study alteration of the white matter network and cerebello-cerebral disconnection that might account for cognitive impairments. The aim of this study was to reconstruct cerebellar WM tracts and to assess the sensitivity of diffusion parameters in detecting the sub-voxel organization of cerebellar afferent and efferent WM fibers associated with unilateral focal cerebellar damage. According to this model, we expect that a degeneration of the cerebellar white matter tracts may ultimately damage the related cerebral GM areas. In order to validate this model, cerebral GM patterns were also investigated to assess the impact of unilateral cerebellar lesion on brain structures. Altogether, these analyses may provide in vivo information about the pathological processes that affect the cortico-cerebellar interaction mechanisms and account for functional alterations observed in cerebellar patients.

## 2. Materials and methods

### 2.1 Subjects

Nine patients with a unilateral cerebellar lesion attending the Specialist Rehabilitation Clinic of Santa Lucia Foundation (Rome, Italy) were recruited for the current study between 2012 and 2016. All patients suffered from an isolated event (ischemic or surgical) which selectively involved the cerebellar parenchyma. The patients were enrolled at least 30 days after the acute event [26] and their neurological symptomatology was fully stabilized. Information about the lesion location (ensuring that patients met the inclusion criteria) was available from previous clinical scans. Nevertheless, in order to minimize any potential bias, the anatomical distribution of tissue damage in terms of unilaterality, cerebellar structures involved, and the absence of any extra-cerebellar pathology were further investigated by an expert neuro-radiologist and performed by visual inspection of the T2-weighted MRI scans acquired as part of this research study. All patients had a unilateral lesion in the left side (Cb-L) [F/M = 4/5; mean age ± SD = 44,8± 13.3 years]. With respect to lesion etiology, 3 patients suffered from a post-surgical lesion (Cb-4; Cb-7; Cb-8), while the remaining ones were diagnosed with an ischemic or hemorrhagic cerebellar stroke. All patients underwent a comprehensive neurological examination, and motor deficits were assessed using the International Cooperative Ataxia Rating Scale (ICARS) [27] whose global score ranges from 0 (absence of any motor deficit) to 100 (presence of motor deficits at the highest degree). Main demographic and clinical characteristics of the patients are reported in Table 1.

**Table 1:** Main demographic and clinical characteristics of the patients.

Case Code	Age	Gender	Lesion Type	ICARS TOTAL SCORES
**Cb-1**	38	F	Ischemic	31.5
**Cb-2**	58	M	Ischemic	9.5
**Cb-3**	52	F	Ischemic	3
**Cb-4**	53	M	Surgical	28
**Cb-5**	44	M	Ischemic	16.5
**Cb-6**	36	M	Ischemic	2
**Cb-7**	62	M	Surgical	7
**Cb-8**	18	F	Surgical	3
**Cb-9**	43	F	Ischemic	46

The table reports for each patient age, gender, lesion etiology and the motor total scores as assessed by the International Cooperative Ataxia Rating Scale (ICARS)

A group of 25 healthy subjects (HS) [F/M = 19/6] ranging from 40 to 60 years of age [mean age ± SD = 53.8 ± 5.9 years] with no history of neurological or psychiatric illness were also recruited for the study as controls.

This research study was approved by the Ethics Committee of Santa Lucia Foundation, according to the principles expressed in the Declaration of Helsinki. Written informed consent was obtained from each subject before study initiation.

### 2.2 MRI acquisition protocol

All subjects underwent an MRI examination at 3T (Magnetom Allegra, Siemens, Erlangen, Germany), including the following acquisitions: 1) dual-echo turbo spin echo [TSE] (TR = 6190 ms, TE = 12/109 ms; Matrix = 192 × 256×48, in-plane FOV = 154×205 mm^2^, slice thickness = 3 mm); 2) fast-FLAIR (TR = 8170 ms, 204TE = 96 ms, TI = 2100 ms; Matrix = 192 × 256×48, in-plane FOV = 154×205 mm^2^, slice thickness = 3 mm); 3) 3D T1-weighted Modified Driven Equilibrium Fourier Transform (MDEFT) scan (TR = 1338 ms, TE = 2.4 ms, Matrix = 256 × 224×176, in-plane FOV = 250×250mm2, slice thickness = 1 mm); 4) diffusion weighted Spin-Echo Echo Planar Imaging (SE EPI) along 61 non-collinear directions (TR = 7 s, TE = 85 ms, b factor = 1000 s.mm^-2^, 45 contiguous slices volumes with a 2.3mm^3^ isotropic reconstructed voxel size). TSE scans were reviewed to exclude the presence of macroscopic brain abnormalities.

### 2.3 Lesion assessment

For each patient, a detailed assessment of the macroscopic cerebellar lesion was performed on high-resolution T1-weighted images. The cerebellum was normalized separately to the Spatially Unbiased Atlas Template of the cerebellum and brainstem (SUIT) [28]. Each lesion was manually outlined using the FSL view image viewer from the FMRIB software library (FSL, www.fmrib.ox.ac.uk/fsl/) and anatomically localized with reference to the SUIT atlas.

### 2.4 MRI imaging and data analyses

#### 2.4.1 DTI processing

Due to the fact that the cerebellum is notorious for having pulsatile artifacts from nearby pulsating blood vessels, we first examined visually the raw diffusion data (and subsequently also the DTI maps) for any such degradation and found the data quality not to be noticeably affected by this kind of artifacts.

Correction for eddy currents and small head movements was done on DTI volumes by means of affine registration to the first non-diffusion weighted volume using FSL [29]. After brain segmentation with the Brain Extraction Tool (BET) utility [30], the diffusion tensor (DT) coefficients were computed in Camino [31] to generate whole brain maps of the DTI metrics including fractional anisotropy (FA), radial diffusivity (RD), mean diffusivity (MD) and axial diffusivity (AD). Each FA volume was registered to the native space MDEFT volume with a linear registration first, followed by a non-linear transformation. The target for the linear registration was the skull-stripped MDEFT, while the original volume (including skull) was the target for the non-linear transformation. The registration was achieved using the tools FLIRT [32] and FNIRT [33] from FSL. This “FA to T1” transformation was combined with each individual “T1 to MNI” transformation, obtained by non-linear registration of the T1 volume to the ICBM152 MNI template. This resulted in the final transformation from each participant’s DTI space to the ICBM152 MNI template.

#### 2.4.2 DTI based tractography

**Fig 1:**
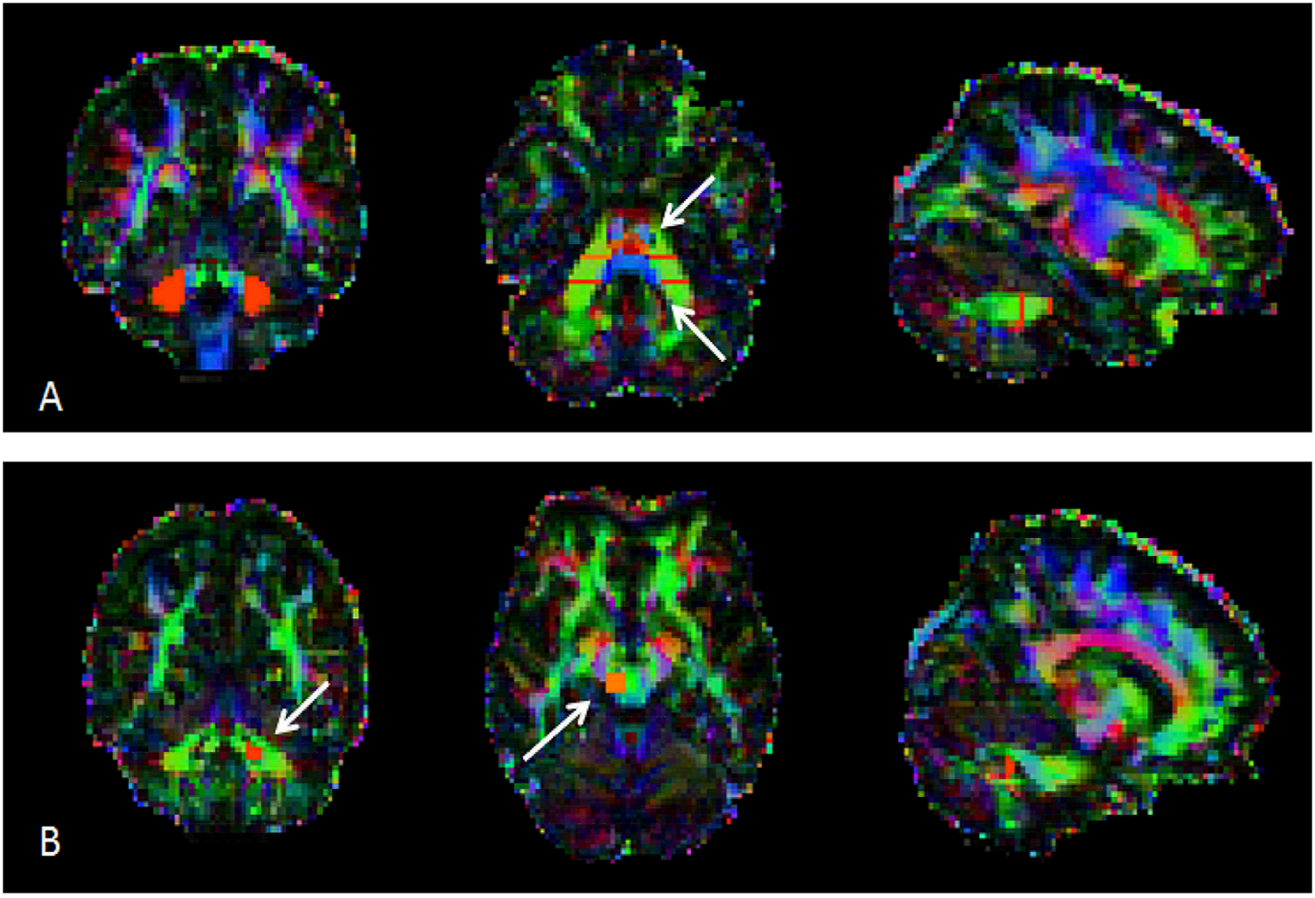
Anatomical localization of cerebellar ROIs for tractography of MCP and SCP. Cerebellar ROIs manually drawn on the FA map images for MCP (A) and SCP (B) tracking. For MCP, the coronal seed ROIs (in red) are illustrated. In the axial slice, cerebellar seed and waypoint ROIs are indicated by the white arrows. For the left SCP the seed region (coronal slice) and the endpoint ROI (axial slice) are illustrated (white arrows). Note that for the right SCP the same ROIs were used swapping right and left hemispheres (ROIs not shown).

MCP and SCP were reconstructed using tractography based on two multi-fiber models implemented in Camino [24]. Q-Ball imaging [34] was used for MCP, as it provides less false positive fiber components. Persistent angular structure (PAS) MRI [35] was used for SCP, due to its ability to describe crossing fibers. Once the multi-fiber directions were estimated, probabilistic tractography was carried out based on the data using the PICo algorithm. N = 10000 tracking iterations were performed from each voxel of the seed Region of Interest (ROI) with stopping criteria of FA ≤ 0:1 and curving angle ≤ 80°. Five ROIs were manually drawn on the FA map images for MCP tracking. A seed ROI was placed bilaterally on a single coronal section anteriorly to the dentate nucleus of each cerebellar hemisphere, and two coronal waypoint ROIs were located bilaterally and anteriorly to each seed ROIs. Finally an exclusion ROI was placed in the axial plane above the pons to prevent fibers not belonging to the middle cerebellar peduncle from being tracked. Left (L-) and right (R-) SCPs were separately reconstructed. For L-SCP, originating from the left cerebellar hemisphere, five ROIs were drawn: one ROI (i.e., “seed” region for tractography) was drawn on a single coronal slice in the dentate nucleus, while two endpoint ROIs were drawn as target points. The first was located posteriorly to the seed to select all extremities going posteriorly, while the second one controlaterally to include the red nucleus and its medial area, where SCP decussates before terminating in the contralateral ventrolateral (VL) nucleus of the thalamus [36–37]. Finally, in order to exclude fibers not belonging to the L-SCP, two ROIs were drawn as exclusion masks and located as follows: the first one was placed immediately superiorly to the second endpoint ROI on the whole coronal slice, and the second one in a sagittal plane to extend superiorly up to a few voxels below the known SCP decussation. The same procedure was followed for the R-SCP, swapping right and left hemispheres. Cerebellar ROIs for reconstruction of MCP and SCP are illustrated in Fig 1. In order to obtain a binary map of the “average tract”, every subject’s reconstructed MCP, L-SCP and R-SCP maps were binarized using a probability threshold for probability index of connectivity (PICo) maps computed by in-house software to minimize the amount of tract volume variation with PICo threshold. These images were then warped into standard space using the FA to ICBM152 MNI space transformation previously calculated, and averaged. Finally, a threshold value has been then applied to the resulting maps in order to retain only those voxels that were common to at least 50% of subjects.

### 2.5 Statistical analysis

#### 2.5.1 Voxel-wise analysis of white matter

A voxel-wise analysis was performed in order to compare diffusivity white matter changes between patients and healthy controls, restricting the comparison to the voxels of the MCP and SCP, based on the average tract masks obtained as described above and similarly to [25]. T-contrasts were evaluated with voxel significance set at p < 0.0001 and corrected for family-wise error (FWE) at cluster level with significance level chosen for p < 0.05. Multiple diffusion tensor measures were used in order to better characterize the tissue microstructure [38]. To remove the effect of confounding variables, the analysis was adjusted for age, since statistically significant differences was found between patients and controls (p = .01). Although there was no difference in gender distribution between groups (*X*^2^ = 3,01, df = 1, p = 0.08), sex was also set as covariate.

#### 2.5.2 Voxel-wise analysis of cerebral GM

T1 volumes were segmented into grey matter (GM) maps and registered to MNI space by means of the “New Segment” and “DARTEL” routines in SPM8 (http://www.fil.ion.ucl.ac.uk/spm/), Wellcome Trust Centre for Neuroimaging, Institute of Neurology, University College London, UK) [39]. VBM statistical analysis was performed to compare the GM maps between the group of patients and healthy subjects entered as independent groups. The analysis excluded voxels in the cerebellum and was restricted to the cerebrum entered as explicit mask. As described above, age, gender and intracranial volume (ICV) were set as covariates of no interest.

T-contrasts were evaluated with voxel significance set at p < 0.0001 and corrected for family-wise error (FWE) at cluster level with significance level chosen for p < 0.05.

## 3.Results

### 3.1 Cerebellar lesion assessment

The cerebellar lesion distribution is summarized, for each patient, in Table 2 (case code as in Table 1). Left Cerebellar hemisphere was affected in most of the patients (8/9) while 6 out of 9 patients presented an involvement of the peduncles: MCP was damaged in Cb-2, Cb-5 and Cb-9, SCP in Cb-3 and Cb-4, and both MCP and SCP were damaged in Cb-1.

**Table 2:** Characteristics of the cerebellar lesion in studied patients.

Case code	Hem	Lobules										DN	vermis	MPC	SCP
		I-IV	V	VI	Crus I	Crus II	VIIB	VIIIA	VIIIB	IX	X				
**Cb-1**	**x**	**x**	**x**	**x**										**x**	**x**
**Cb-2**	**x**		**x**	**x**								**x**		**x**	
**Cb-3**															**x**
**Cb-4**	**x**	**x**	**x**	**x**								**x**			**x**
**Cb-5**	**x**	**x**												**x**	
**Cb-6**	**x**		**x**	**x**	**x**										
**Cb-7**	**x**		**x**	**x**	**x**										
**Cb-8**	**x**						**x**	**x**		**x**			**x**		
**Cb-9**	**x**	**x**	**x**	**x**	**x**					**x**				**x**	

The extension of the lesion (X) as depicted in the MRI reports is here summarized for each patient. Case code as in Table 1. Table Legend: Hem: cerebellar hemispheres; DN: dentate nucleus; MCP: middle cerebellar peduncle; SCP: superior cerebellar peduncle.

**Fig 2:**
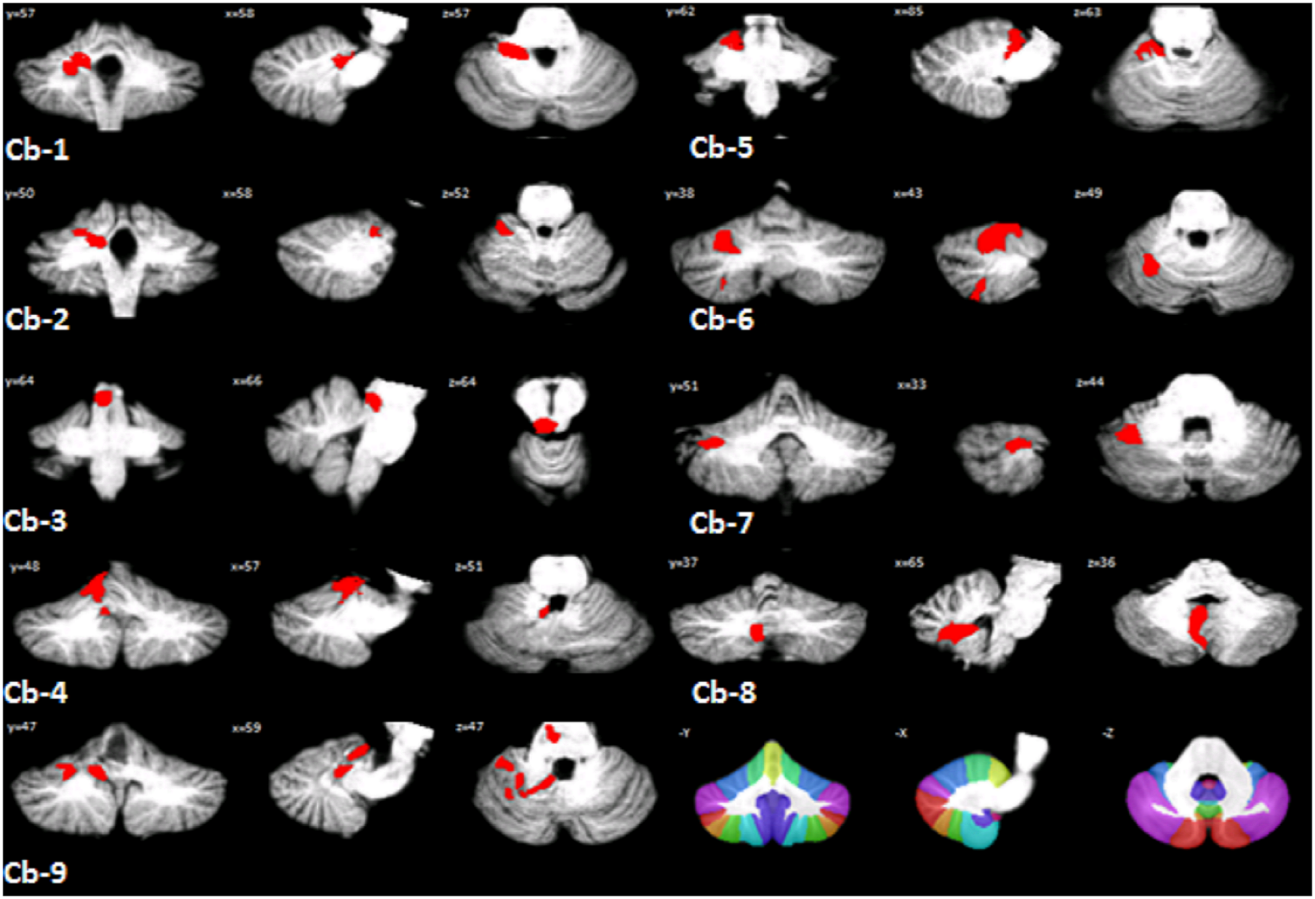
Lesion reconstruction and distribution in patients. Each individual lesion is presented and superimposed on coronal (= y), sagittal (= x) and axial (= z) slices of the SUIT atlas template (Diedrichsen et al., 2009) after spatial normalization. Patients codes as in Tables 1 and 2. The bottom left of the figure shows the SUIT atlas.

Specifically, cerebellar lesion distribution for each patient is illustrated in Fig 2 and summarized in Table 2. Note that, lobules I, II, III, and IV are combined in the SUIT atlas and they are referred to as Lobules I–IV.

Additionally, the overall white matter difference was assessed between patients and controls. As showed by the t-test analysis, no differences between groups were detected in terms of total cerebral white matter volume (p = .99).

### 3.2 White matter tract analysis

**Fig 3:**
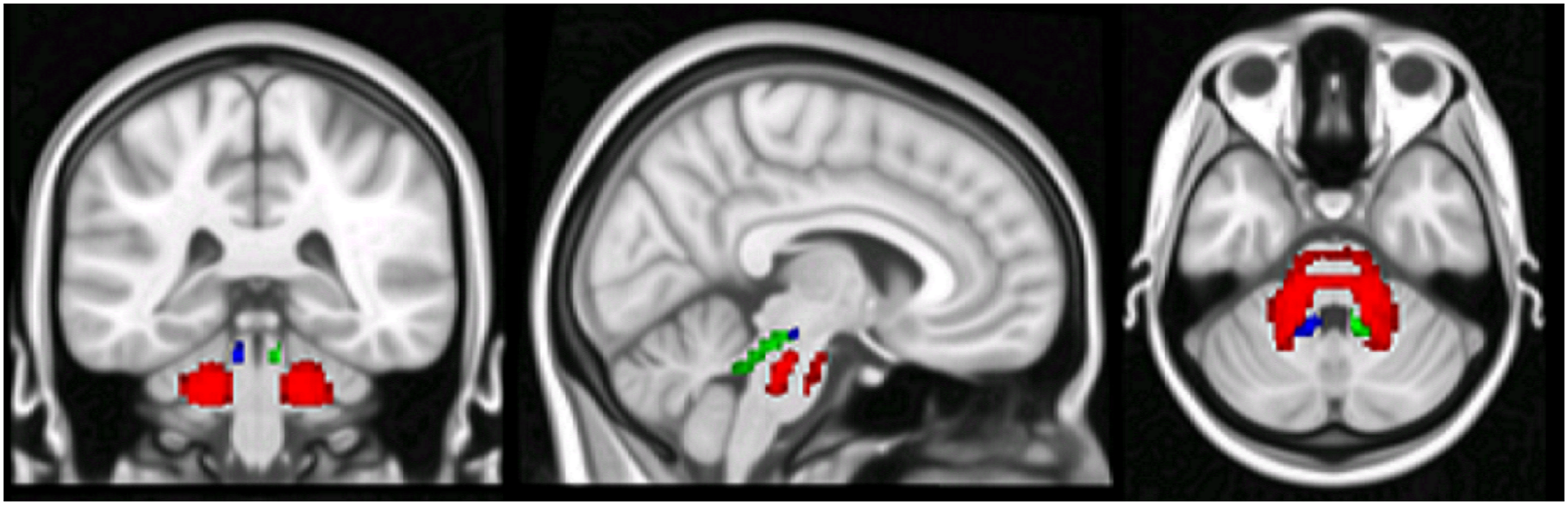
DTI-based tractography of middle and superior cerebellar peduncles. DTI-based tractography of the average tract of MCP (red), L-SCP (blue) and R-SCP (green) with voxels belonging to at least 50% of the subjects.

**Fig 4:**
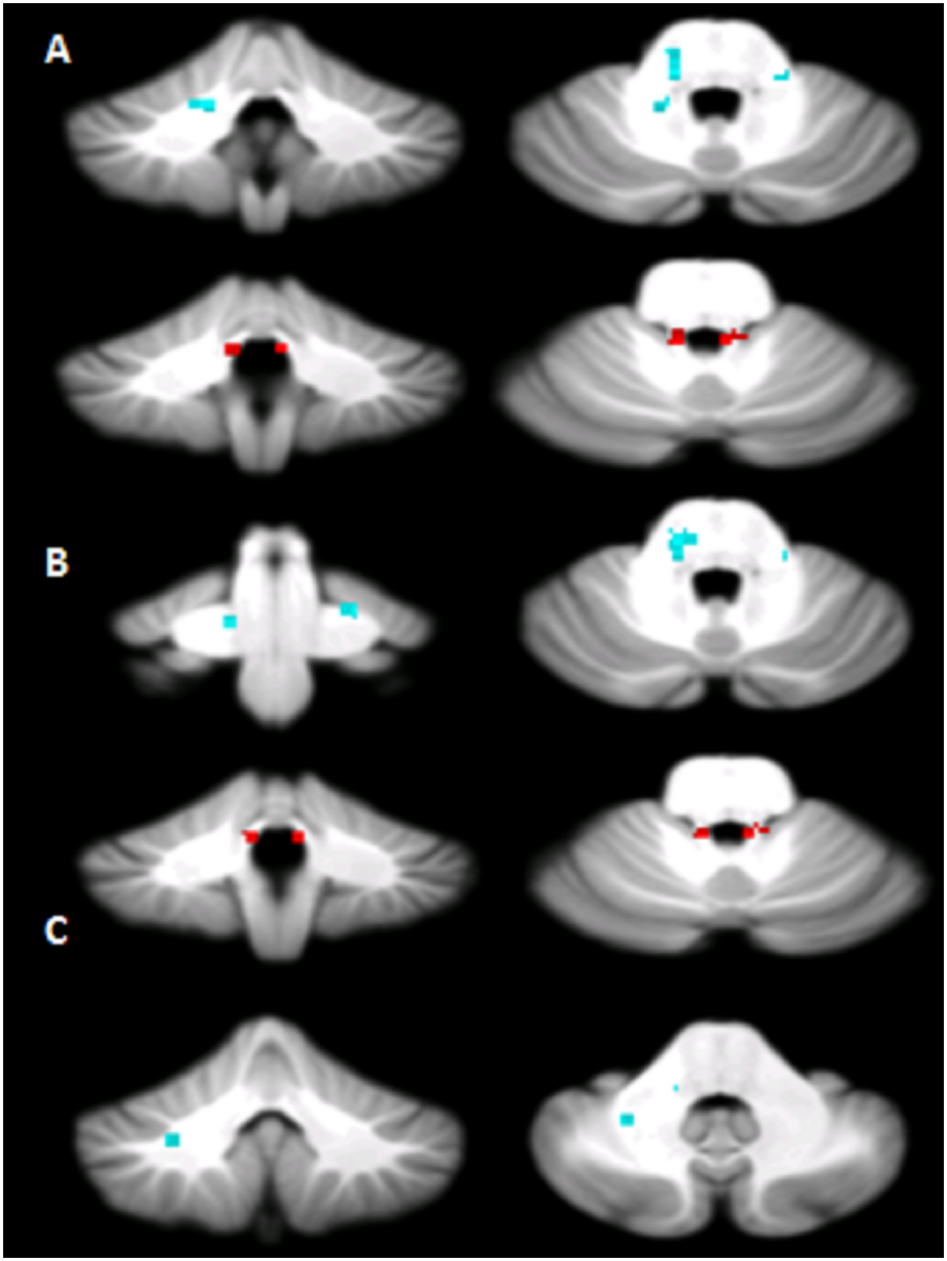
Voxel-wise analysis of white matter tracts. Regions showing altered Radial Diffusivity (A), Mean Diffusivity (B) and Fractional anisotropy (C) in patients compared to controls. The regions in the middle cerebellar peduncle are shown in light blue; the regions in superior cerebellar peduncle are shown in red. Axial diffusivity was not significantly affected (data not shown).

MCP and SCP were successfully reconstructed in patients and HS (See S1 File). Fig 3 shows the fiber reconstruction for the average MCP and SCP of both groups of subjects. Voxel-wise comparisons between patients and HS were performed for each diffusion metric separately. WM analysis showed RD and MD to increase both in ipsilateral MCP and SCP of patients compared to HS, while FA to decrease only in ipsilateral MCP. Interestingly, contralateral MCP and SCP were also found to show an increase of RD and MD, while no significant effect on FA was observed. Finally, AD appeared to be not significantly affected either ipsilaterally or controlaterally. Results are illustrated in Fig 4.(See also S1 File). Detailed statistics of cerebellar white-matter voxel-wise comparisons are summarized in Table 3.

**Table 3:** Statistics of cerebellar white-matter voxel-wise comparisons for each patients’ group.

		Side	Size (NoV)	Coordinates(mm)			Peak Z-scores
				X	Z	Y	
**RD (Cb-L>HS)**	**MCP**	L	13	-10	-34	-30	**3.92**
	**SCP**	L	16	-6	-44	26	**4.53**
		R	9	6	44	26	**3.78**
**MD (Cb-L>HS)**	**MCP**	L	12	-16	-48	-28	**4.21**
	**SCP**	L	13	-6	-44	26	**4.33**
		R	6	6	-44	26	**3.70**
**FA (Cb-L<HS)**	**MCP**	L	8	-12	-40	-34	**3.70**
	**SCP**	L	-	-	-	-	-
		R	-	-	-	-	-

Regions of significant diffusivity white matter changes between patients and healthy controls restricting the comparison to the voxels of the MCP and SCP. Altered diffusion tensor measures are reported separately for each tract. Only regions that survived after correction for multiple comparisons (FWE corrected p <0.05) have been considered. Stereotaxic coordinates are reported in MNI space. NoV = Number of voxels in the cluster.

### 3.3 VBM analysis across groups

**Fig 5:**
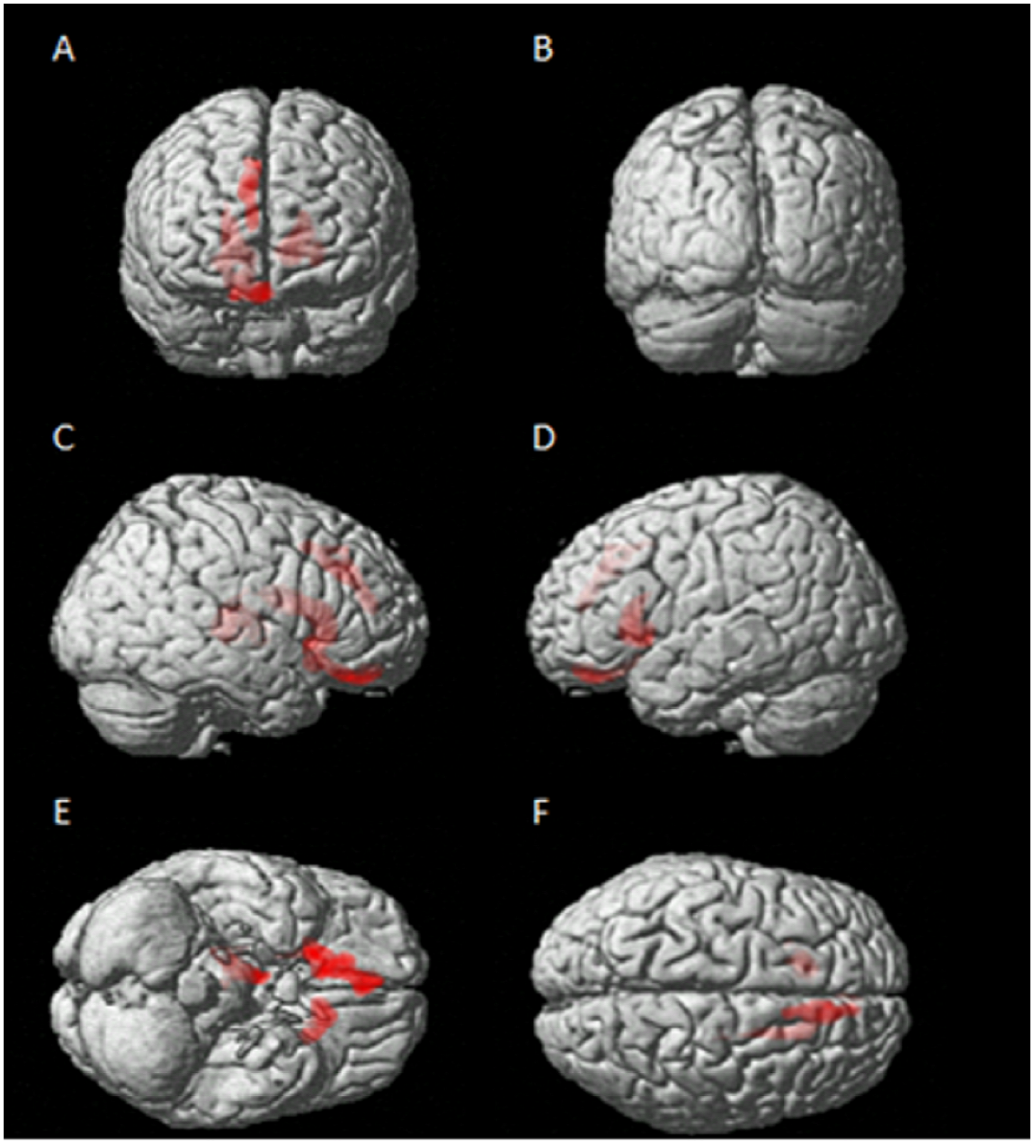
Between groups voxel-based comparison of cerebral GM density. Regions showing patterns of reduced regional GM in patients compared to HS in both contralateral and ipsilateral cerebral cortex.

None of the considered T1 volume scans were affected by macroscopic artifacts as assessed by visual examination. Comparisons were performed between patient group and HS. Compared to HS, patients showed a widespread pattern of regional GM loss, which involved both cerebral hemispheres. Specifically, a pattern of GM loss was found to affect the contralateral thalamus, caudate, orbitofrontal and paracingulate cortices. Additionally, ipsilateral caudate and putamen were found to show a pattern of GM loss. These results are illustrated in Fig 5 (See also S2 File). Detailed statistics of the whole brain voxel-wise comparisons are summarized in Table 4.

**Table 4:** Statistics of whole brain voxel-wise comparisons for each patients’ group (Cb-L<HS).

Brain Region	Side	Size(NoV)	Coordinates(mm)			Peak Z-scores
			X	Y	Z	
**Frontal Orbital**	R	1876	18	18	-14	**4.33**
**Caudate**	L	897	-9	24	3	**5.19**
**Putamen**	L		-22	18	-2	**3.91**
**Thalamus**	R	784	18	-19	9	**6.27**
**Paracingulate Gyrus**	R	696	11	32	33	**4.92**
			9	44	19	**3.89**
			11	15	46	**3.65**

The regions of significantly decreased grey matter density in patients with left cerebellar lesion compared to healthy controls are reported. Only regions that survived after correction for multiple comparisons (FWE corrected p <0.05) have been considered. Stereotaxic coordinates are reported in MNI space.

## 4. Discussion

In the present study we aimed at reconstructing MCP and SCP and describing the pattern of white matter alterations associated with unilateral cerebellar lesions. We performed diffusion-based tractography to assess the sub-voxel organization/disruption of these tracts. Previous DTI studies have identified and isolated the cerebellar projections to prefrontal and parietal cortices in healthy subjects [20–22], and have shown specific RD changes in the cerebellar peduncles of patients with hereditary or sporadic cerebellar ataxia [40–41, 25]. To our knowledge, this is the first study to examine the structural pattern of MCP and SCP in patients with unilateral cerebellar lesions. In terms of integrity, white matter architecture of MCP and SCP showed a specific pattern of diffusion changes. The most intriguing finding was that, in spite of the unilaterality of the lesion, microstructural changes (where by microstructural we mean with no corresponding abnormalities visible on conventional scan) were present bilaterally in MCP and SCP at least at group level. This is more interesting taking into account that macroscopic abnormalities (i.e., visible lesions) were present only unilaterally on MRI scans of all subjects. Multiple diffusion parameters were analyzed and selective diffusivity changes were detected. Overall, an increase of RD and MD without significant changes in AD was found in both MCP and SCP bilaterally with FA significantly decreased only in the ipsilesional MCP. Increased MD and decreased FA have been typically reported in chronic ischemic lesions (> 2 weeks) [38, 42]. This is consistent with our sample; 6 out of 9 patients presented a cerebellar chronic ischemic lesion. On the other hand RD and AD provide information on myelin and axon conditions. Specifically myelination affects RD, [20, 43–44], while axonal damage affects AD. Thus, our findings indicate prevalent bilateral myelin damage with relative axonal sparing. Focal cerebellar lesions have been described to result in impaired higher cognitive functions, associated with structural modifications in cerebral cortex regions functionally linked to the cerebellar lesioned areas [6, 12]. Specifically, a focal cerebellar lesion has been described to result in a functional impairment of the contralateral cerebral cortex (crossed cerebello-cerebral diaschisis) [14–15, 45–46], consistent with the prominent anatomical properties of the cerebello-cerebral projections that for the most part are crossed [6, 10]. However, in the present study cerebral regions ipsilateral to the lesioned cerebellum, namely caudate nucleus and putamen, were also found to show significant GM alterations. Similar ipsilesional changes have also been observed in the cerebral cortex in a previous VBM study [12]. Bilateral cerebellar influences over cerebral cortex are also supported by lesional studies in rodents showing abnormal activity in the ipsilesional sensorimotor cortex [47] not to mention that ipsilateral connections between cerebellum and cerebral cortex have also been shown [48–49]. In light of this evidence, it is reasonable to hypothesize that not only contralateral but also ipsilateral networks may suffer from a unilateral damage of the cerebellum. Present findings, indicating changes in the cerebellar peduncles sub-voxel structure bilaterally in face of a unilateral cerebellar lesion, may impact our understating of cerebro-cerebellar interplay. As most of the recruited patients had a lesion in the left cerebellum, we cannot conclude with certainty that a right-side lesion would result in the same pattern of damage. Further investigations are needed to confirm that these findings can be generalized.

Different arguments can be put forward to explain this result. Firstly considering the extensive literature, cited above, indicating that a unilateral cerebellar lesion may functionally and structurally affect cerebral cortex bilaterally, it might be hypothesized that the bilateral cortical impairment may rebound on the functionality of the lesion-free cerebellum inducing the observed myelin peduncle damages.

On the other hand, resting state functional MRI (fMRI) studies widely also demonstrated functional coherence between the two cerebellar hemispheres [50–52] and dentate nuclei [49]. Thus, a bilateral cerebellar coupling may also explain the myelin damage contralateral to the lesion side. In support of this theory the existence of a cerebellar commissural system has been suggested by anatomical data [49, 53–54].

However, another possibility has to be considered. Bilateral microstructural alterations in the peduncles might be related to closeness to the ischemic lesion. Indeed, a different grade of structural alterations in perilesional chronic ischemic areas is a well-known phenomenon [55]. The DTI alterations observed contralaterally to the RMN ischemic lesion, such as increased RD and MD, is partially consistent with a possible chronic microstructural ischemic pattern as well as a chronic demyelination [20, 38, 56].

Overall, although the precise mechanism inducing bilateral abnormalities in MCP and SCP is still unclear, its presence questions the interpretation of functional and structural alterations observed in the cerebral cortex after unilateral damage of the cerebellar efferents.

A limitation of the study that needs to be discussed is that, due to the small sample of patients recruited, direct correlation between GM changes, WM structural alterations and cognitive performances was not attempted. However, it should be considered that the strict inclusion criteria clearly affected the recruitment rate. In spite of these limitations, present results highly support the hypothesis of a bilateral cerebello-cortical functional whose confirmation will require further studies involving larger populations of patients. This will also allow to explore the relationship between brain characteristics and clinical outcomes of patients, as well as to better establish the direct causality between GM changes and WM structural alterations. To conclude, present findings indicate that, in face of a unilateral cerebellar lesion, bilateral changes in the cerebellar peduncles microstructure can be observed. Consistently, cerebral GM reduction can be found without lateralization. Altogether, these structural observations may provide important insights into understanding cerebro-cerebellar interaction in health and disease. This latter aspect is of particular value considering the increasing interest of cerebellar neuromodulation to treat different CNS disease [57–59].

## Supporting information

S1 File (WM_Voxel_Wise_Analysis) Supporting MRI data file contains the averaged MCP, L-SCP and R-SCP used as explicit masks and T-contrast and results of voxel-wise analysis between cerebellar patients and HS for FA, MD, and RD within the reconstructed tracts. (ZIP)

S2 File (GM_Voxel_Based_Morphometry) Supporting MRI data file contains T-contrast and results of cerebral GM voxel-based morphometry between cerebellar patients and HS. (ZIP)
